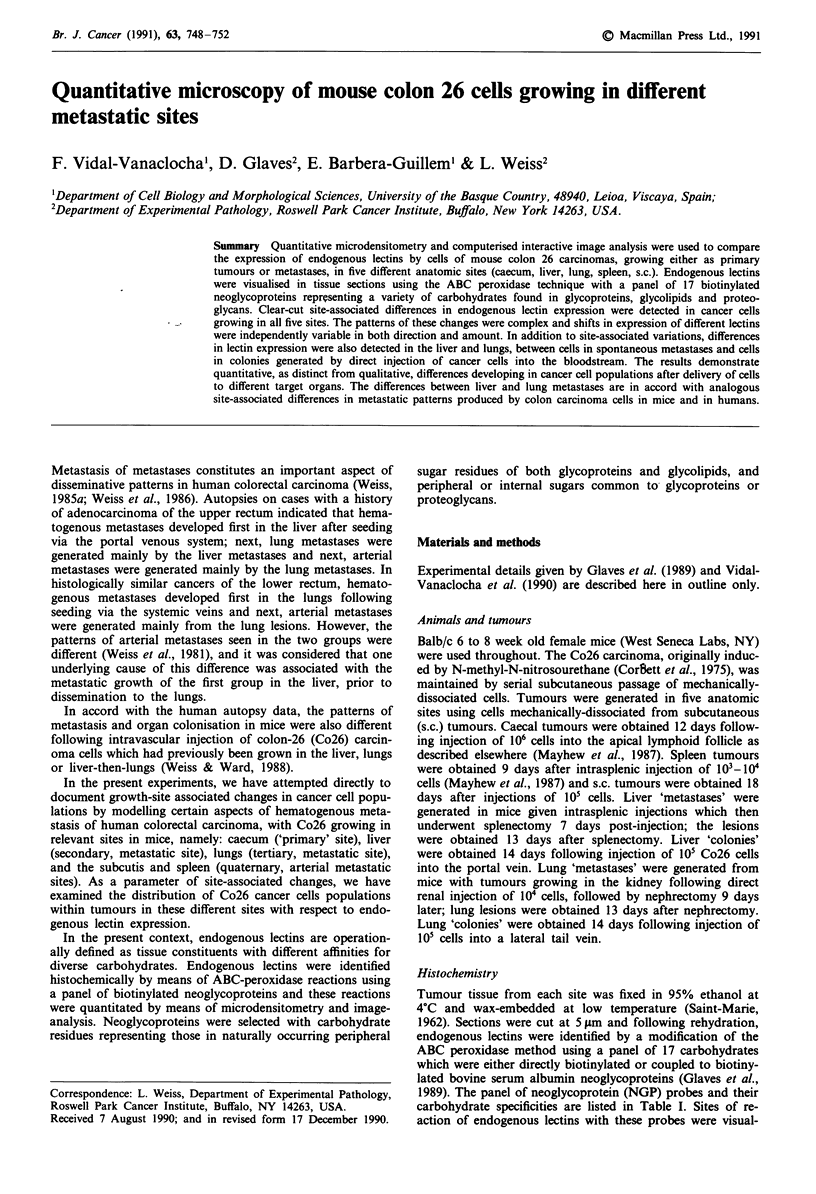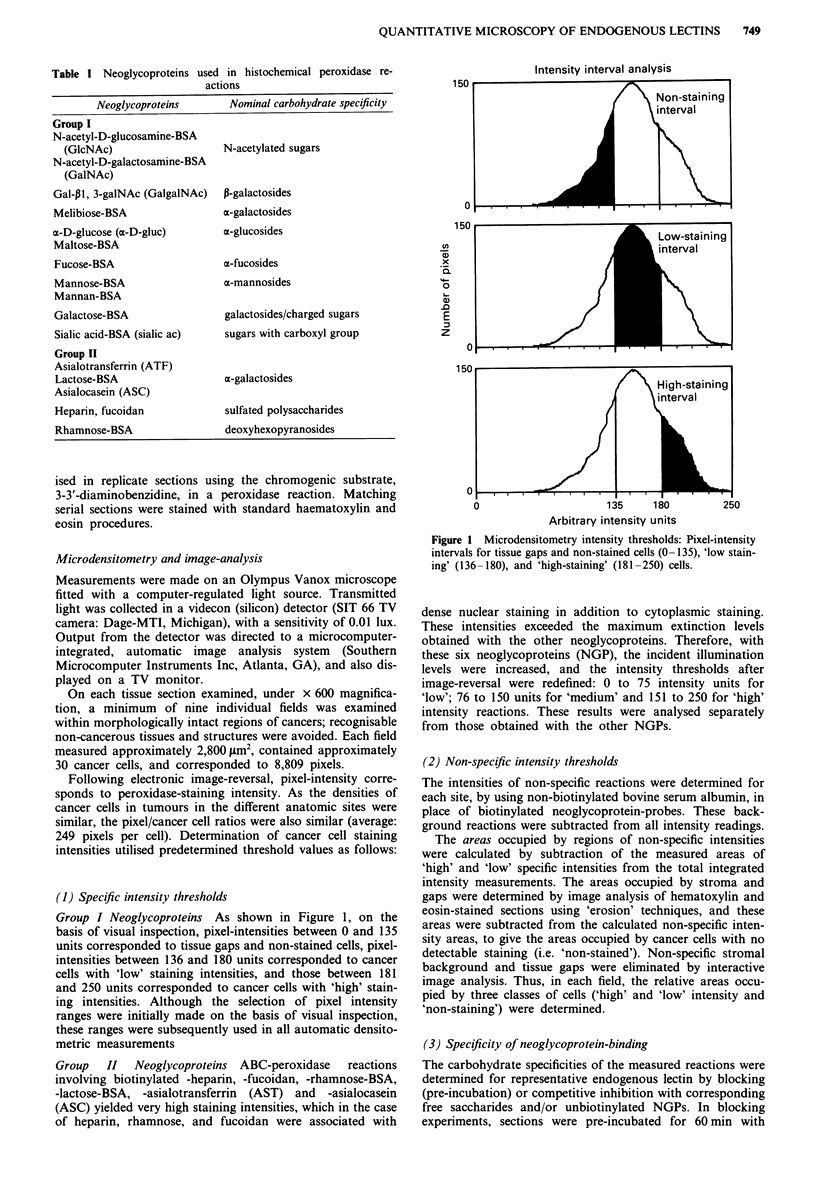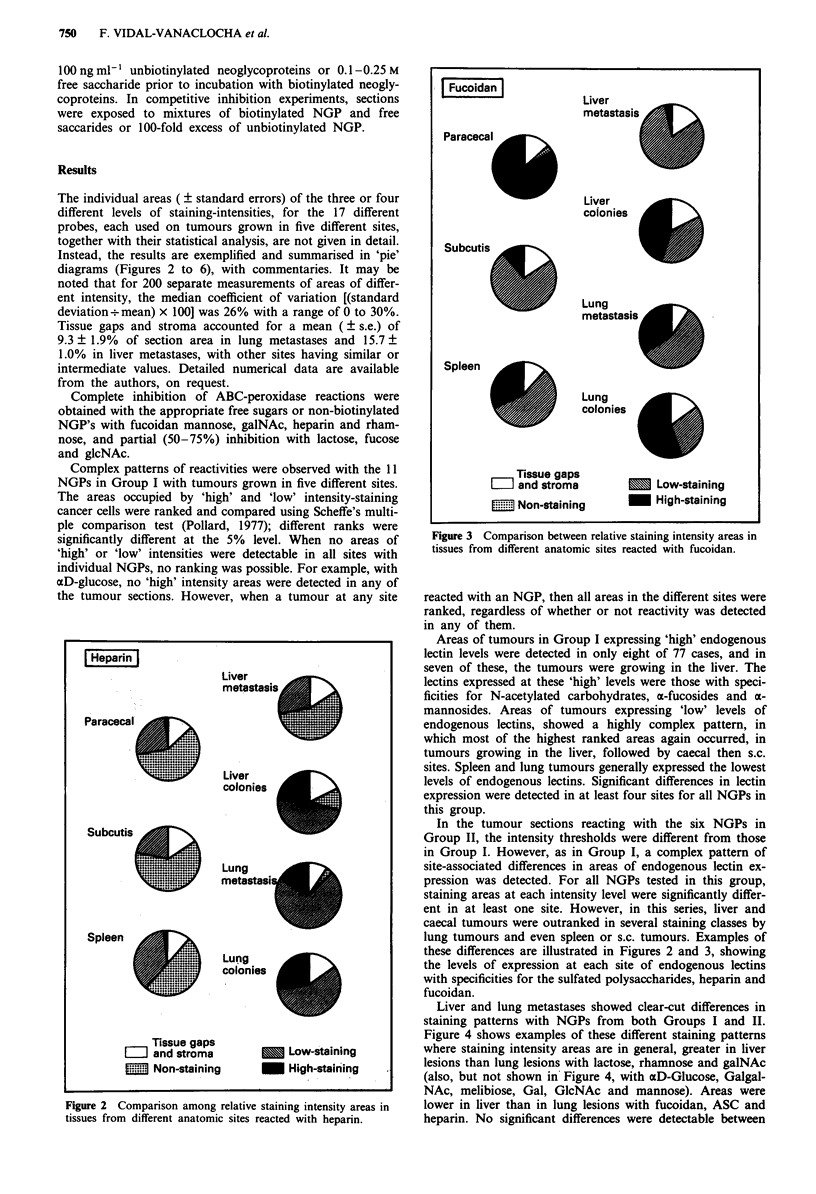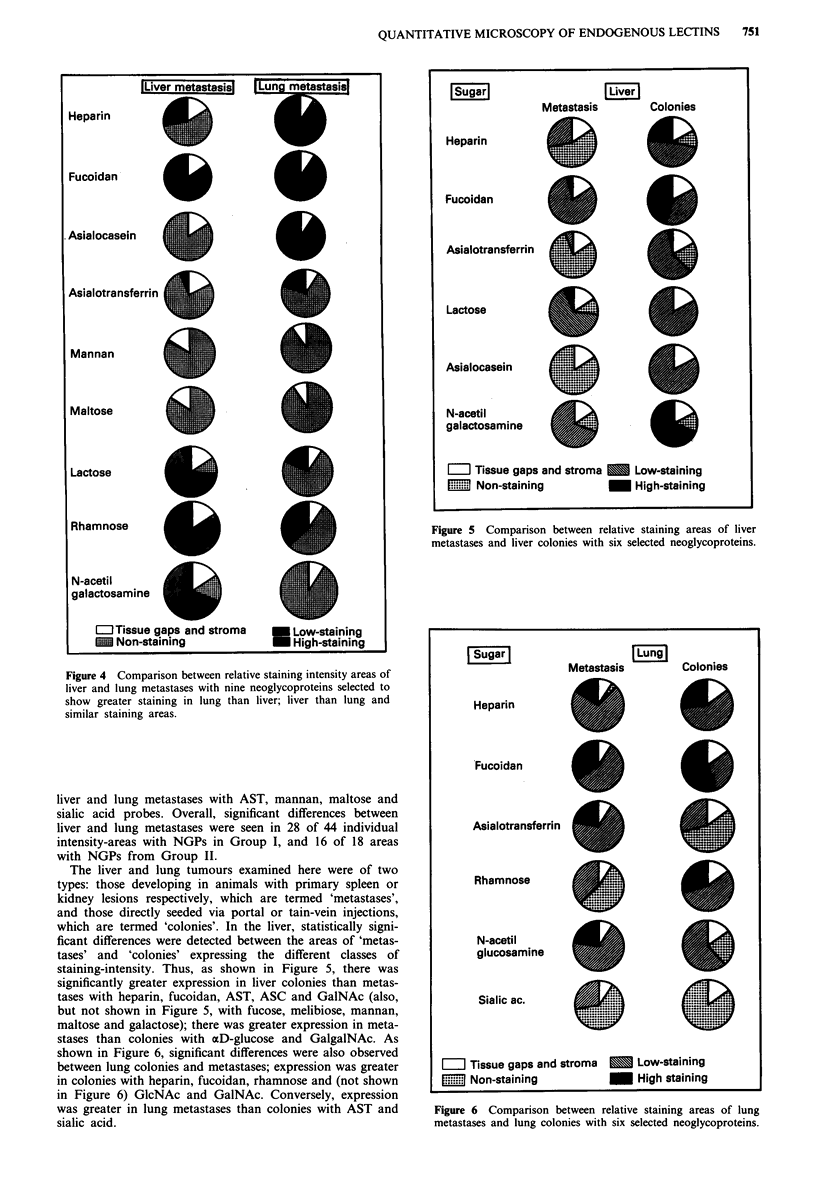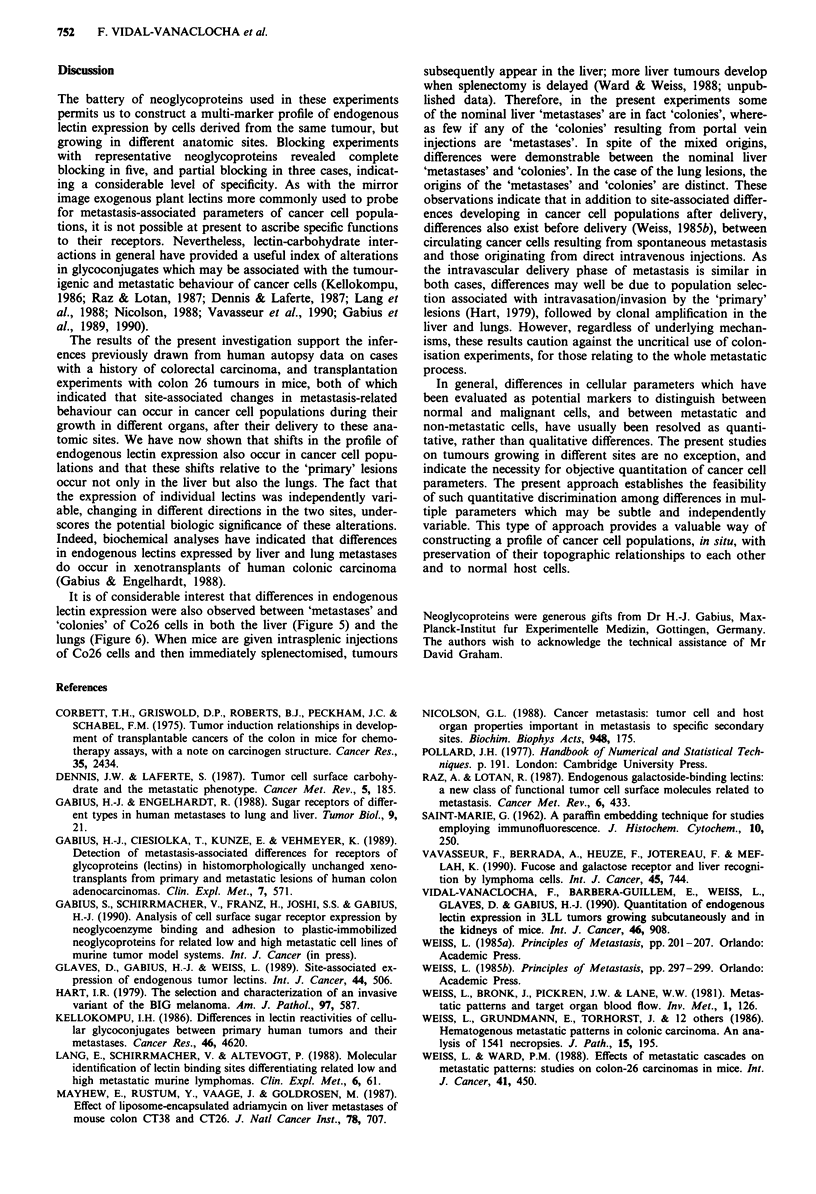# Quantitative microscopy of mouse colon 26 cells growing in different metastatic sites.

**DOI:** 10.1038/bjc.1991.167

**Published:** 1991-05

**Authors:** F. Vidal-Vanaclocha, D. Glaves, E. Barbera-Guillem, L. Weiss

**Affiliations:** Department of Cell Biology and Morphological Sciences, University of the Basque Country, Leioa, Viscaya, Spain.

## Abstract

Quantitative microdensitometry and computerised interactive image analysis were used to compare the expression of endogenous lectins by cells of mouse colon 26 carcinomas, growing either as primary tumours or metastases, in five different anatomic sites (caecum, liver, lung, spleen, s.c.). Endogenous lectins were visualised in tissue sections using the ABC peroxidase technique with a panel of 17 biotinylated neoglycoproteins representing a variety of carbohydrates found in glycoproteins, glycolipids and proteoglycans. Clear-cut site-associated differences in endogenous lectin expression were detected in cancer cells growing in all five sites. The patterns of these changes were complex and shifts in expression of different lectins were independently variable in both direction and amount. In addition to site-associated variations, differences in lectin expression were also detected in the liver and lungs, between cells in spontaneous metastases and cells in colonies generated by direct injection of cancer cells into the bloodstream. The results demonstrate quantitative, as distinct from qualitative, differences developing in cancer cell populations after delivery of cells to different target organs. The differences between liver and lung metastases are in accord with analogous site-associated differences in metastatic patterns produced by colon carcinoma cells in mice and in humans.


Br. J. Cancer (1991), 63, 748-752 © Macmillan Press Ltd., 1991

### Quantitative microscopy of mouse colon 26 cells growing in different metastatic sites

F. Vidal-Vanaclocha[1], D. Glaves[2], E. Barbera-Guillem[1] & L. Weiss[2]

[1]*Department of Cell Biology and Morphological Sciences, University of the Basque Country, 48940, Leioa, Viscaya, Spain;* [2]*Department of Experimental Pathology, Roswell Park Cancer Institute, Buffalo, New York 14263, USA.*

**Summary** Quantitative microdensitometry and computerised interactive image analysis were used to compare the expression of endogenous lectins by cells of mouse colon 26 carcinomas, growing either as primary tumours or metastases, in five different anatomic sites (caecum, liver, lung, spleen, s.c.). Endogenous lectins were visualised in tissue sections using the ABC peroxidase technique with a panel of 17 biotinylated neoglycoproteins representing a variety of carbohydrates found in glycoproteins, glycolipids and proteoglycans. Clear-cut site-associated differences in endogenous lectin expression were detected in cancer cells growing in all five sites. The patterns of these changes were complex and shifts in expression of different lectins were independently variable in both direction and amount. In addition to site-associated variations, differences in lectin expression were also detected in the liver and lungs, between cells in spontaneous metastases and cells in colonies generated by direct injection of cancer cells into the bloodstream. The results demonstrate quantitative, as distinct from qualitative, differences developing in cancer cell populations after delivery of cells to different target organs. The differences between liver and lung metastases are in accord with analogous site-associated differences in metastatic patterns produced by colon carcinoma cells in mice and in humans.

Metastasis of metastases constitutes an important aspect of disseminative patterns in human colorectal carcinoma (Weiss, 1985*a*; Weiss *et al.*, 1986). Autopsies on cases with a history of adenocarcinoma of the upper rectum indicated that hematogenous metastases developed first in the liver after seeding via the portal venous system; next, lung metastases were generated mainly by the liver metastases and next, arterial metastases were generated mainly by the lung metastases. In histologically similar cancers of the lower rectum, hematogenous metastases developed first in the lungs following seeding via the systemic veins and next, arterial metastases were generated mainly from the lung lesions. However, the patterns of arterial metastases seen in the two groups were different (Weiss *et al.*, 1981), and it was considered that one underlying cause of this difference was associated with the metastatic growth of the first group in the liver, prior to dissemination to the lungs.

In accord with the human autopsy data, the patterns of metastasis and organ colonisation in mice were also different following intravascular injection of colon-26 (Co26) carcinoma cells which had previously been grown in the liver, lungs or liver-then-lungs (Weiss & Ward, 1988).

In the present experiments, we have attempted directly to document growth-site associated changes in cancer cell populations by modelling certain aspects of hematogenous metastasis of human colorectal carcinoma, with Co26 growing in relevant sites in mice, namely: caecum ('primary' site), liver (secondary, metastatic site), lungs (tertiary, metastatic site), and the subcutis and spleen (quaternary, arterial metastatic sites). As a parameter of site-associated changes, we have examined the distribution of Co26 cancer cells populations within tumours in these different sites with respect to endogenous lectin expression.

In the present context, endogenous lectins are operationally defined as tissue constituents with different affinities for diverse carbohydrates. Endogenous lectins were identified histochemically by means of ABC-peroxidase reactions using a panel of biotinylated neoglycoproteins and these reactions were quantitated by means of microdensitometry and image-analysis. Neoglycoproteins were selected with carbohydrate residues representing those in naturally occurring peripheral sugar residues of both glycoproteins and glycolipids, and peripheral or internal sugars common to glycoproteins or proteoglycans.

### Materials and methods

Experimental details given by Glaves *et al.* (1989) and Vidal-Vanaclocha *et al.* (1990) are described here in outline only.

### *Animals and tumours*

Balb/c 6 to 8 week old female mice (West Seneca Labs, NY) were used throughout. The Co26 carcinoma, originally induced by N-methyl-N-nitrosourethane (Corbett *et al.*, 1975), was maintained by serial subcutaneous passage of mechanically-dissociated cells. Tumours were generated in five anatomic sites using cells mechanically-dissociated from subcutaneous (s.c.) tumours. Caecal tumours were obtained 12 days following injection of $10^6$ cells into the apical lymphoid follicle as described elsewhere (Mayhew *et al.*, 1987). Spleen tumours were obtained 9 days after intrasplenic injection of $10^3-10^4$ cells (Mayhew *et al.*, 1987) and s.c. tumours were obtained 18 days after injections of $10^5$ cells. Liver 'metastases' were generated in mice given intrasplenic injections which then underwent splenectomy 7 days post-injection; the lesions were obtained 13 days after splenectomy. Liver 'colonies' were obtained 14 days following injection of $10^5$ Co26 cells into the portal vein. Lung 'metastases' were generated from mice with tumours growing in the kidney following direct renal injection of $10^4$ cells, followed by nephrectomy 9 days later; lung lesions were obtained 13 days after nephrectomy. Lung 'colonies' were obtained 14 days following injection of $10^5$ cells into a lateral tail vein.

### *Histochemistry*

Tumour tissue from each site was fixed in 95% ethanol at 4°C and wax-embedded at low temperature (Saint-Marie, 1962). Sections were cut at 5 μm and following rehydration, endogenous lectins were identified by a modification of the ABC peroxidase method using a panel of 17 carbohydrates which were either directly biotinylated or coupled to biotinylated bovine serum albumin neoglycoproteins (Glaves *et al.*, 1989). The panel of neoglycoprotein (NGP) probes and their carbohydrate specificities are listed in Table I. Sites of reaction of endogenous lectins with these probes were visual-

Correspondence: L. Weiss, Department of Experimental Pathology, Roswell Park Cancer Institute, Buffalo, NY 14263, USA.
Received 7 August 1990; and in revised form 17 December 1990.

**Table I** Neoglycoproteins used in histochemical peroxidase reactions

| *Neoglycoproteins* | *Nominal carbohydrate specificity* |
|---|---|
| **Group I** | |
| N-acetyl-D-glucosamine-BSA (GlcNAc) | N-acetylated sugars |
| N-acetyl-D-galactosamine-BSA (GalNAc) | |
| Gal-$\beta$1, 3-galNAc (GalgalNAc) | $\beta$-galactosides |
| Melibiose-BSA | $\alpha$-galactosides |
| $\alpha$-D-glucose ($\alpha$-D-gluc) Maltose-BSA | $\alpha$-glucosides |
| Fucose-BSA | $\alpha$-fucosides |
| Mannose-BSA Mannan-BSA | $\alpha$-mannosides |
| Galactose-BSA | galactosides/charged sugars |
| Sialic acid-BSA (sialic ac) | sugars with carboxyl group |
| **Group II** | |
| Asialotransferrin (ATF) Lactose-BSA Asialocasein (ASC) | $\alpha$-galactosides |
| Heparin, fucoidan | sulfated polysaccharides |
| Rhamnose-BSA | deoxyhexopyranosides |

ised in replicate sections using the chromogenic substrate, 3-3'-diaminobenzidine, in a peroxidase reaction. Matching serial sections were stained with standard haematoxylin and eosin procedures.

### *Microdensitometry and image-analysis*

Measurements were made on an Olympus Vanox microscope fitted with a computer-regulated light source. Transmitted light was collected in a videcon (silicon) detector (SIT 66 TV camera: Dage-MTI, Michigan), with a sensitivity of 0.01 lux. Output from the detector was directed to a microcomputer-integrated, automatic image analysis system (Southern Microcomputer Instruments Inc, Atlanta, GA), and also displayed on a TV monitor.

On each tissue section examined, under × 600 magnification, a minimum of nine individual fields was examined within morphologically intact regions of cancers; recognisable non-cancerous tissues and structures were avoided. Each field measured approximately 2,800 $\mu m^2$, contained approximately 30 cancer cells, and corresponded to 8,809 pixels.

Following electronic image-reversal, pixel-intensity corresponds to peroxidase-staining intensity. As the densities of cancer cells in tumours in the different anatomic sites were similar, the pixel/cancer cell ratios were also similar (average: 249 pixels per cell). Determination of cancer cell staining intensities utilised predetermined threshold values as follows:

### *(1) Specific intensity thresholds*

*Group I Neoglycoproteins* As shown in Figure 1, on the basis of visual inspection, pixel-intensities between 0 and 135 units corresponded to tissue gaps and non-stained cells, pixel-intensities between 136 and 180 units corresponded to cancer cells with 'low' staining intensities, and those between 181 and 250 units corresponded to cancer cells with 'high' staining intensities. Although the selection of pixel intensity ranges were initially made on the basis of visual inspection, these ranges were subsequently used in all automatic densitometric measurements

*Group II Neoglycoproteins* ABC-peroxidase reactions involving biotinylated -heparin, -fucoidan, -rhamnose-BSA, -lactose-BSA, -asialotransferrin (AST) and -asialocasein (ASC) yielded very high staining intensities, which in the case of heparin, rhamnose, and fucoidan were associated with

**Figure 1** Microdensitometry intensity thresholds: Pixel-intensity intervals for tissue gaps and non-stained cells (0–135), 'low staining' (136–180), and 'high-staining' (181–250) cells.

dense nuclear staining in addition to cytoplasmic staining. These intensities exceeded the maximum extinction levels obtained with the other neoglycoproteins. Therefore, with these six neoglycoproteins (NGP), the incident illumination levels were increased, and the intensity thresholds after image-reversal were redefined: 0 to 75 intensity units for 'low'; 76 to 150 units for 'medium' and 151 to 250 for 'high' intensity reactions. These results were analysed separately from those obtained with the other NGPs.

### *(2) Non-specific intensity thresholds*

The intensities of non-specific reactions were determined for each site, by using non-biotinylated bovine serum albumin, in place of biotinylated neoglycoprotein-probes. These background reactions were subtracted from all intensity readings.

The *areas* occupied by regions of non-specific intensities were calculated by subtraction of the measured areas of 'high' and 'low' specific intensities from the total integrated intensity measurements. The areas occupied by stroma and gaps were determined by image analysis of hematoxylin and eosin-stained sections using 'erosion' techniques, and these areas were subtracted from the calculated non-specific intensity areas, to give the areas occupied by cancer cells with no detectable staining (i.e. 'non-stained'). Non-specific stromal background and tissue gaps were eliminated by interactive image analysis. Thus, in each field, the relative areas occupied by three classes of cells ('high' and 'low' intensity and 'non-staining') were determined.

### *(3) Specificity of neoglycoprotein-binding*

The carbohydrate specificities of the measured reactions were determined for representative endogenous lectin by blocking (pre-incubation) or competitive inhibition with corresponding free saccharides and/or unbiotinylated NGPs. In blocking experiments, sections were pre-incubated for 60 min with

100 ng ml$^{-1}$ unbiotinylated neoglycoproteins or 0.1–0.25 M free saccharide prior to incubation with biotinylated neoglycoproteins. In competitive inhibition experiments, sections were exposed to mixtures of biotinylated NGP and free saccharides or 100-fold excess of unbiotinylated NGP.

### Results

The individual areas ( ± standard errors) of the three or four different levels of staining-intensities, for the 17 different probes, each used on tumours grown in five different sites, together with their statistical analysis, are not given in detail. Instead, the results are exemplified and summarised in 'pie' diagrams (Figures 2 to 6), with commentaries. It may be noted that for 200 separate measurements of areas of different intensity, the median coefficient of variation [(standard deviation ÷ mean) × 100] was 26% with a range of 0 to 30%. Tissue gaps and stroma accounted for a mean ( ± s.e.) of 9.3 ± 1.9% of section area in lung metastases and 15.7 ± 1.0% in liver metastases, with other sites having similar or intermediate values. Detailed numerical data are available from the authors, on request.

Complete inhibition of ABC-peroxidase reactions were obtained with the appropriate free sugars or non-biotinylated NGP's with fucoidan mannose, galNAc, heparin and rhamnose, and partial (50–75%) inhibition with lactose, fucose and glcNAc.

Complex patterns of reactivities were observed with the 11 NGPs in Group I with tumours grown in five different sites. The areas occupied by 'high' and 'low' intensity-staining cancer cells were ranked and compared using Scheffe's multiple comparison test (Pollard, 1977); different ranks were significantly different at the 5% level. When no areas of 'high' or 'low' intensities were detectable in all sites with individual NGPs, no ranking was possible. For example, with αD-glucose, no 'high' intensity areas were detected in any of the tumour sections. However, when a tumour at any site reacted with an NGP, then all areas in the different sites were ranked, regardless of whether or not reactivity was detected in any of them.

**Figure 3** Comparison between relative staining intensity areas in tissues from different anatomic sites reacted with fucoidan.

**Figure 2** Comparison among relative staining intensity areas in tissues from different anatomic sites reacted with heparin.

Areas of tumours in Group I expressing 'high' endogenous lectin levels were detected in only eight of 77 cases, and in seven of these, the tumours were growing in the liver. The lectins expressed at these 'high' levels were those with specificities for N-acetylated carbohydrates, α-fucosides and α-mannosides. Areas of tumours expressing 'low' levels of endogenous lectins, showed a highly complex pattern, in which most of the highest ranked areas again occurred, in tumours growing in the liver, followed by caecal then s.c. sites. Spleen and lung tumours generally expressed the lowest levels of endogenous lectins. Significant differences in lectin expression were detected in at least four sites for all NGPs in this group.

In the tumour sections reacting with the six NGPs in Group II, the intensity thresholds were different from those in Group I. However, as in Group I, a complex pattern of site-associated differences in areas of endogenous lectin expression was detected. For all NGPs tested in this group, staining areas at each intensity level were significantly different in at least one site. However, in this series, liver and caecal tumours were outranked in several staining classes by lung tumours and even spleen or s.c. tumours. Examples of these differences are illustrated in Figures 2 and 3, showing the levels of expression at each site of endogenous lectins with specificities for the sulfated polysaccharides, heparin and fucoidan.

Liver and lung metastases showed clear-cut differences in staining patterns with NGPs from both Groups I and II. Figure 4 shows examples of these different staining patterns where staining intensity areas are in general, greater in liver lesions than lung lesions with lactose, rhamnose and galNAc (also, but not shown in Figure 4, with αD-Glucose, Galgal-NAc, melibiose, Gal, GlcNAc and mannose). Areas were lower in liver than in lung lesions with fucoidan, ASC and heparin. No significant differences were detectable between

**Figure 4** Comparison between relative staining intensity areas of liver and lung metastases with nine neoglycoproteins selected to show greater staining in lung than liver; liver than lung and similar staining areas.

liver and lung metastases with AST, mannan, maltose and sialic acid probes. Overall, significant differences between liver and lung metastases were seen in 28 of 44 individual intensity-areas with NGPs in Group I, and 16 of 18 areas with NGPs from Group II.

The liver and lung tumours examined here were of two types: those developing in animals with primary spleen or kidney lesions respectively, which are termed 'metastases', and those directly seeded via portal or tain-vein injections, which are termed 'colonies'. In the liver, statistically significant differences were detected between the areas of 'metastases' and 'colonies' expressing the different classes of staining-intensity. Thus, as shown in Figure 5, there was significantly greater expression in liver colonies than metastases with heparin, fucoidan, AST, ASC and GalNAc (also, but not shown in Figure 5, with fucose, melibiose, mannan, maltose and galactose); there was greater expression in metastases than colonies with αD-glucose and GalgalNAc. As shown in Figure 6, significant differences were also observed between lung colonies and metastases; expression was greater in colonies with heparin, fucoidan, rhamnose and (not shown in Figure 6) GlcNAc and GalNAc. Conversely, expression was greater in lung metastases than colonies with AST and sialic acid.

**Figure 5** Comparison between relative staining areas of liver metastases and liver colonies with six selected neoglycoproteins.

**Figure 6** Comparison between relative staining areas of lung metastases and lung colonies with six selected neoglycoproteins.

### Discussion

The battery of neoglycoproteins used in these experiments permits us to construct a multi-marker profile of endogenous lectin expression by cells derived from the same tumour, but growing in different anatomic sites. Blocking experiments with representative neoglycoproteins revealed complete blocking in five, and partial blocking in three cases, indicating a considerable level of specificity. As with the mirror image exogenous plant lectins more commonly used to probe for metastasis-associated parameters of cancer cell populations, it is not possible at present to ascribe specific functions to their receptors. Nevertheless, lectin-carbohydrate interactions in general have provided a useful index of alterations in glycoconjugates which may be associated with the tumourigenic and metastatic behaviour of cancer cells (Kellokompu, 1986; Raz & Lotan, 1987; Dennis & Laferte, 1987; Lang *et al.*, 1988; Nicolson, 1988; Vavasseur *et al.*, 1990; Gabius *et al.*, 1989, 1990).

The results of the present investigation support the inferences previously drawn from human autopsy data on cases with a history of colorectal carcinoma, and transplantation experiments with colon 26 tumours in mice, both of which indicated that site-associated changes in metastasis-related behaviour can occur in cancer cell populations during their growth in different organs, after their delivery to these anatomic sites. We have now shown that shifts in the profile of endogenous lectin expression also occur in cancer cell populations and that these shifts relative to the 'primary' lesions occur not only in the liver but also the lungs. The fact that the expression of individual lectins was independently variable, changing in different directions in the two sites, underscores the potential biologic significance of these alterations. Indeed, biochemical analyses have indicated that differences in endogenous lectins expressed by liver and lung metastases do occur in xenotransplants of human colonic carcinoma (Gabius & Engelhardt, 1988).

It is of considerable interest that differences in endogenous lectin expression were also observed between 'metastases' and 'colonies' of Co26 cells in both the liver (Figure 5) and the lungs (Figure 6). When mice are given intrasplenic injections of Co26 cells and then immediately splenectomised, tumours subsequently appear in the liver; more liver tumours develop when splenectomy is delayed (Ward & Weiss, 1988; unpublished data). Therefore, in the present experiments some of the nominal liver 'metastases' are in fact 'colonies', whereas few if any of the 'colonies' resulting from portal vein injections are 'metastases'. In spite of the mixed origins, differences were demonstrable between the nominal liver 'metastases' and 'colonies'. In the case of the lung lesions, the origins of the 'metastases' and 'colonies' are distinct. These observations indicate that in addition to site-associated differences developing in cancer cell populations after delivery, differences also exist before delivery (Weiss, 1985*b*), between circulating cancer cells resulting from spontaneous metastasis and those originating from direct intravenous injections. As the intravascular delivery phase of metastasis is similar in both cases, differences may well be due to population selection associated with intravasation/invasion by the 'primary' lesions (Hart, 1979), followed by clonal amplification in the liver and lungs. However, regardless of underlying mechanisms, these results caution against the uncritical use of colonisation experiments, for those relating to the whole metastatic process.

In general, differences in cellular parameters which have been evaluated as potential markers to distinguish between normal and malignant cells, and between metastatic and non-metastatic cells, have usually been resolved as quantitative, rather than qualitative differences. The present studies on tumours growing in different sites are no exception, and indicate the necessity for objective quantitation of cancer cell parameters. The present approach establishes the feasibility of such quantitative discrimination among differences in multiple parameters which may be subtle and independently variable. This type of approach provides a valuable way of constructing a profile of cancer cell populations, *in situ*, with preservation of their topographic relationships to each other and to normal host cells.

Neoglycoproteins were generous gifts from Dr H.-J. Gabius, Max-Planck-Institut fur Experimentelle Medizin, Gottingen, Germany. The authors wish to acknowledge the technical assistance of Mr David Graham.

### References

CORBETT, T.H., GRISWOLD, D.P., ROBERTS, B.J., PECKHAM, J.C. & SCHABEL, F.M. (1975). Tumor induction relationships in development of transplantable cancers of the colon in mice for chemotherapy assays, with a note on carcinogen structure. *Cancer Res.*, **35**, 2434.

DENNIS, J.W. & LAFERTE, S. (1987). Tumor cell surface carbohydrate and the metastatic phenotype. *Cancer Met. Rev.*, **5**, 185.

GABIUS, H.-J. & ENGELHARDT, R. (1988). Sugar receptors of different types in human metastases to lung and liver. *Tumor Biol.*, **9**, 21.

GABIUS, H.-J., CIESIOLKA, T., KUNZE, E. & VEHMEYER, K. (1989). Detection of metastasis-associated differences for receptors of glycoproteins (lectins) in histomorphologically unchanged xenotransplants from primary and metastatic lesions of human colon adenocarcinomas. *Clin. Expl. Met.*, **7**, 571.

GABIUS, S., SCHIRRMACHER, V., FRANZ, H., JOSHI, S.S. & GABIUS, H.-J. (1990). Analysis of cell surface sugar receptor expression by neoglycoenzyme binding and adhesion to plastic-immobilized neoglycoproteins for related low and high metastatic cell lines of murine tumor model systems. *Int. J. Cancer* (in press).

GLAVES, D., GABIUS, H.-J. & WEISS, L. (1989). Site-associated expression of endogenous tumor lectins. *Int. J. Cancer*, **44**, 506.

HART, I.R. (1979). The selection and characterization of an invasive variant of the BIG melanoma. *Am. J. Pathol.*, **97**, 587.

KELLOKOMPU, I.H. (1986). Differences in lectin reactivities of cellular glycoconjugates between primary human tumors and their metastases. *Cancer Res.*, **46**, 4620.

LANG, E., SCHIRRMACHER, V. & ALTEVOGT, P. (1988). Molecular identification of lectin binding sites differentiating related low and high metastatic murine lymphomas. *Clin. Expl. Met.*, **6**, 61.

MAYHEW, E., RUSTUM, Y., VAAGE, J. & GOLDROSEN, M. (1987). Effect of liposome-encapsulated adriamycin on liver metastases of mouse colon CT38 and CT26. *J. Natl Cancer Inst.*, **78**, 707.

NICOLSON, G.L. (1988). Cancer metastasis: tumor cell and host organ properties important in metastasis to specific secondary sites. *Biochim. Biophys Acts*, **948**, 175.

POLLARD, J.H. (1977). *Handbook of Numerical and Statistical Techniques*. p. 191. London: Cambridge University Press.

RAZ, A. & LOTAN, R. (1987). Endogenous galactoside-binding lectins: a new class of functional tumor cell surface molecules related to metastasis. *Cancer Met. Rev.*, **6**, 433.

SAINT-MARIE, G. (1962). A paraffin embedding technique for studies employing immunofluorescence. *J. Histochem. Cytochem.*, **10**, 250.

VAVASSEUR, F., BERRADA, A., HEUZE, F., JOTEREAU, F. & MEFLAH, K. (1990). Fucose and galactose receptor and liver recognition by lymphoma cells. *Int. J. Cancer*, **45**, 744.

VIDAL-VANACLOCHA, F., BARBERA-GUILLEM, E., WEISS, L., GLAVES, D. & GABIUS, H.-J. (1990). Quantitation of endogenous lectin expression in 3LL tumors growing subcutaneously and in the kidneys of mice. *Int. J. Cancer*, **46**, 908.

WEISS, L. (1985*a*). *Principles of Metastasis*, pp. 201–207. Orlando: Academic Press.

WEISS, L. (1985*b*). *Principles of Metastasis*, pp. 297–299. Orlando: Academic Press.

WEISS, L., BRONK, J., PICKREN, J.W. & LANE, W.W. (1981). Metastatic patterns and target organ blood flow. *Inv. Met.*, **1**, 126.

WEISS, L., GRUNDMANN, E., TORHORST, J. & 12 others (1986). Hematogenous metastatic patterns in colonic carcinoma. An analysis of 1541 necropsies. *J. Path.*, **15**, 195.

WEISS, L. & WARD, P.M. (1988). Effects of metastatic cascades on metastatic patterns: studies on colon-26 carcinomas in mice. *Int. J. Cancer*, **41**, 450.